# Home Remodeling and Food Allergy Interact Synergistically to Increase the Risk of Atopic Dermatitis

**DOI:** 10.1155/2017/3793679

**Published:** 2017-09-20

**Authors:** Won Seok Lee, Kyung Suk Lee, Shinhae Lee, Myongsoon Sung, Seung-Jin Lee, Hye Mi Jee, Youn Ho Sheen, Man Yong Han, Young-Ho Jung

**Affiliations:** ^1^Department of Pediatrics, Graduate School, Kyung Hee University, Seoul, Republic of Korea; ^2^Department of Pediatrics, CHA Bundang Medical Center, CHA University School of Medicine, Seongnam, Republic of Korea; ^3^Department of Pediatrics, Haeundae Paik Hospital, Inje University School of Medicine, Busan, Republic of Korea; ^4^Department of Pediatrics, CHA Gangnam Medical Center, CHA University School of Medicine, Seoul, Republic of Korea

## Abstract

**Purpose:**

The purpose of this study was to investigate the effects of home remodeling and food allergy (FA) on the development of atopic dermatitis (AD) in children.

**Methods:**

The Modified International Study of Asthma and Allergies in Childhood questionnaire was used to survey 4,111 children recruited from 3 kindergartens and 6 elementary schools from Seongnam, Korea. Participants' parents agreed for them to participate in physical examinations, skin prick tests, and blood tests.

**Results:**

Home remodeling in the past 12 months (adjusted odds ratio [aOR] 3.40, *P* = 0.006), lifetime diagnosis of FA (aOR 3.95, *P* < 0.001), parental history of AD (aOR 2.67, *P* = 0.001), and FA (aOR 2.35, *P* = 0.004) were independent risk factors for lifetime diagnosis of AD ever. When history of home remodeling and FA were combined, the risk for moderate-to-severe AD by scoring atopic dermatitis (SCORAD) score increased (aOR, 7.19, *P* = 0.011,* P* for interaction = 0.034).

**Conclusion:**

Home remodeling, lifetime diagnosis of FA, parental history of AD, and parental history of FA were independent risk factors for AD. In addition, we observed a synergistic interaction between home remodeling and FA in the risk of moderate-to-severe AD.

## 1. Introduction

Atopic dermatitis (AD) typically arises at an early age and is the most common chronic, relapsing, inflammatory eczematous skin disease [1]. The pathogenesis of AD is complex with multifactorial etiologies involving genetic, immunological, and environmental factors. The prevalence of AD has risen globally in recent years [1]. There are several reasons for this trend, including genetic factors and increasing prevalence of food allergy (FA) [2, 3]. Recently, environmental factors involving air pollution have been considered as a newly emerging cause for the increased prevalence of AD [2].

Indoor air pollution is an important environmental factor for children, as they tend to spend most of their daytime indoors [4]. Materials affecting outdoor air include particulate matter < 10 mm (PM_10_), nitrogen oxides (NO_*x*_), sulfur oxides (SO_*x*_), and ozone (O_3_) [5]. Materials with substantial effect on indoor air pollution are different from outdoor materials [4]. The causative materials of indoor air pollution include wallpaper, flooring, and paint [6]. Formaldehyde, volatile organic compounds (VOCs), and aromatic compounds are pollutants that are considered more important than other indoor chemicals [7]. Mendell reported that indoor pollutants may play a key role in the development and aggravation of allergic diseases such as AD [4]. High concentrations of VOCs or formaldehyde are associated with the development of Sick Building Syndrome (SBS) and the aggravation of allergic diseases in newly built dwellings [8]. In one study, authors have shown that exposure to home renovation was associated with a higher risk of allergic diseases in children [9]. Therefore, it can be inferred that these changes to the indoor environment may play a considerable role in increasing the incidence of AD.

FA is defined as adverse health reactions to foods consisting of any unanticipated reactions following the ingestion of foods or food additives [10]. Previously published literature indicated that FA plays an important role in exacerbating severe forms of AD [11]. Approximately one-third of children with severe AD have also been reported to have IgE mediated FA [12].

Based on these data, we hypothesized that home remodeling and FA may be linked with the development of AD. However, there have been no studies determining that home remodeling and FA together are risk factors for AD in Korea. We sought to examine the effects of home remodeling and FA on the development of AD in children and determine how they contribute synergistically to the occurrence of this disease.

## 2. Materials and Methods

### 2.1. Subjects

This cross-sectional study was based on a population of 5,196 children aged 4–13 years who attended 3 kindergartens and 6 elementary schools in Seongnam, Korea, between June and July, 2015. Of these subjects, 4,111 completed the questionnaire (response rate, 79.1%) [13]. The participants' parents provided consented for their children to participate in a physical examination, skin prick tests (SPTs), and blood sampling. Pediatricians and trained field technicians conducted the physical examinations, SPTs, and blood sampling at the participating schools. Data pertaining parental economic status were collected and converted to US dollars using an exchange rate of US $1 = 1112.40 South Korean won (exchange rate at June, 1, 2015) [14]. Characteristics of the subjects are described in Table 1.

This study was approved by the Institutional Review Board of the CHA Bundang Medical Center. Written consent was obtained from all parents or guardians following a detailed explanation.

### 2.2. Modified International Study of Asthma and Allergies in Childhood Questionnaire

A modified Korean version of the International Study of Asthma and Allergies in Childhood (ISAAC) questionnaire was used to determine the prevalence of symptoms and diagnosis of allergic diseases [13]. The questionnaire was consisted of three main sections: (1) general characteristics including sex, date of birth, height, and weight; (2) a history of symptoms related to asthma, allergic rhinitis (AR), AD, and FA; and (3) exposure to environmental factors, including home remodeling.

A child was deemed to have been diagnosed with AD and FA or to have a history of home remodeling if an affirmative answer was given to questions “has your child ever been diagnosed with AD by a physician?,” “has your child ever been diagnosed with FA by a physician?,” and “have you ever done home remodeling?,” respectively.

### 2.3. Scoring Atopic Dermatitis Index

Three pediatricians (Dr. Jee, Dr. Jung, and Dr. Lee) visited each school and calculated the scoring atopic dermatitis (SCORAD) scores of each participant in an enclosed space at their respective school. The AD group was divided into three classes based on the severity of AD: mild (<25), moderate (25–50), and severe (>50) [15].

### 2.4. Skin Prick Test and Laboratory Test

SPTs were performed on the volar surface of the skin of the arm with normal appearance using standardized allergen extracts and control solutions from Laforma (Milan, Italy). Subjects were tested for sensitivity to the following 22 common allergens:* Dermatophagoides pteronyssinus (D.p.)*,* Dermatophagoides farinae (D.f.),* birch, oak, walnut, apple, peach, kiwi, egg, milk, cod, pork, elm, hops, peanut, wheat, orange, tomato, strawberry, celery, mussel, and shrimp. Subjects were deemed to be atopic if they tested positive to one or more allergen in the SPTs (allergen and histamine wheal diameter > 3 mm) [16]. White blood cell counts were measured, and the percentage of blood eosinophils was calculated.

### 2.5. Statistical Analysis

Statistical analyses were performed using SPSS version 23.0 (IBM Co., Armonk, NY, USA). Prevalence was presented in 95% confidence intervals (CIs). Logistic regression analyses were conducted to identify independent risk factors for AD. Multivariate analysis was adjusted for personal, familial, and socioeconomic factors. To test the interaction effect between environments (home remodeling and FA history) on AD, logistic regression analysis between home remodeling and FA history on AD was performed. For all analyses (two-tailed), *P* < 0.05 was considered to indicate statistical significance.

## 3. Results

### 3.1. Subject Characteristics

The children were aged 8.00 ± 1.85 years. The majority of the participants were boys (51.7%) and approximately 56% of the participants had a parental history of allergic diseases, including asthma, AR, AD, and FA (Table 1).

### 3.2. Prevalence of AD

AD-related prevalence is listed in Table 2. Current AD, defined as lifetime diagnosis together with the presence of symptoms in the past 12 months in the questionnaire, was 11.7%. Moderate-to-severe AD (SCORAD score > 25) was 6.6%.

### 3.3. Risk Factors for Lifetime Diagnosis of AD

Independent risk factors for lifetime diagnosis of AD were: girl (aOR 1.69, *P* = 0.016), lifetime diagnosis of FA (aOR 3.95, *P* < 0.001), lifetime diagnosis of asthma (aOR 3.38, *P* < 0.001), lifetime diagnosis of AR (aOR 2.37, *P* < 0.001), parental history of allergic diseases (aOR 3.22, *P* < 0.001), parental history of AD (aOR 2.67, *P* = 0.001), and home remodeling in the past 12 months (aOR 3.40, *P* = 0.006) (Table 3).

### 3.4. Home Remodeling Increases the Risk of AD

**Figure 1:**
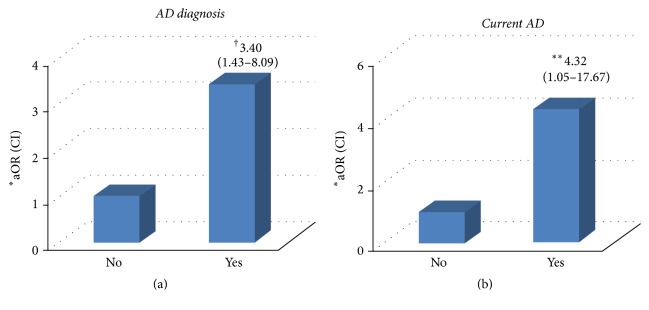
Home remodeling within recent 1 year as a risk factor of lifetime diagnosis of AD (*N* = 123) and current AD (*N* = 100). (a) Home remodeling in the past 12 months is a risk factor of lifetime diagnosis of AD (^†^aOR 3.40, 95% CI 1.43–8.09, *P* = 0.006). (b) Home remodeling in the past 12 months is a risk factor of current AD (^*∗∗*^aOR 4.32, 95% CI 1.05–17.67, *P* = 0.042). The data was calculated by logistic regression multivariate analysis. aOR: adjusted odds ratio; CI: confidence interval; BMI: body mass index; AD: atopic dermatitis. ^*∗*^aOR was adjusted by age, sex, BMI, parental history of AD, familial income, and eosinophil.

Children with a home remodeling history in the past 12 months had an increased risk for lifetime diagnosis of AD (aOR = 3.40, *P* = 0.006) (Figure 1(a)). Children with a home remodeling history in the past 12 months had increased risk for current AD (aOR = 4.32, *P* = 0.042) (Figure 1(b)).

### 3.5. Home Remodeling and FA History Act Synergistically to Increase Risk of Moderate-to-Severe AD

**Figure 2:**
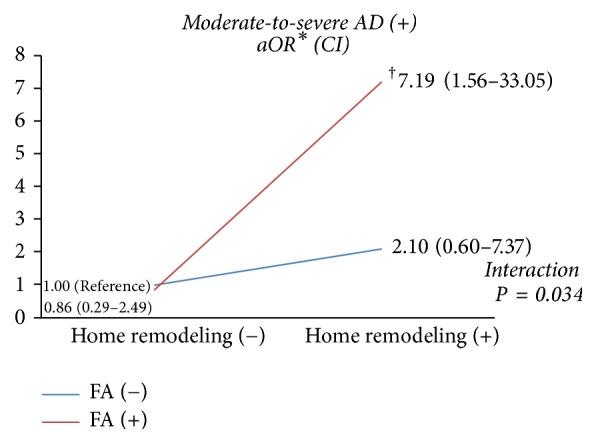
Effect of home remodeling on the risk of moderate-to-severe AD by SCORAD score in children with and without FA history (*N* = 421). When home remodeling and FA variable were combined, the risk for moderate-to-severe AD incidence was significantly increased. (aOR 7.19, 95% CI 1.56–33.05, ^†^*P* = 0.011, *P* for interaction = 0.034). The data was calculated by logistic regression multivariate analysis. aOR: adjusted odds ratio; CI: confidence interval; BMI: body mass index; AD: atopic dermatitis; SCORAD: scoring atopic dermatitis; FA: food allergy. ^*∗*^aOR was adjusted by age, sex, BMI, parental history of AD, familial income, and eosinophil.

Children were divided into four groups based on their history of home remodeling within 12 months and FA. When home remodeling and FA variable were combined, the risk for moderate-to-severe AD incidence was significantly increased (aOR = 7.19, *P* = 0.011, *P* for interaction = 0.034) (Figure 2).

## 4. Discussion

The purpose of this cross-sectional study was to investigate how home remodeling and FA may be associated with AD in children and how they interact with each other with regard to AD. In this study, overall lifetime diagnosis of AD was 30.2%, and several independent risk factors were identified that increased the risk for AD. When lifetime diagnosis of home remodeling and FA were combined, the risk for moderate-to-severe AD significantly increased. Home remodeling history in the past 12 months was also an independent risk factor for current AD.

Housing reconstruction and remodeling activities have rapidly developed in the past several decades in Korea [17]. Many people previously lived in private houses, but during the past several years, they have gradually moved to community housing, such as apartments [17]. Nuclear families have become more common, and married young couples often move to newly built apartments or remodeled houses [17].

A variety of materials are used for reconstruction and house remodeling, including organic solvents, heavy metals, and VOCs, such as benzene, toluene, xylene, styrene, and formaldehyde, may be emitted from paints or dyes [4]. VOCs can damage the epidermal barrier and increase the adverse effects of house dust mites on sensitized subjects with AD [18]. Only short-term exposure to formaldehyde can cause dysfunction of the skin barrier in children with and without AD; this is more prominent in the latter [19]. In an experimental rat model of AD, exposure to formaldehyde aggravated pruritus and dermatitis and was associated with an elevated expression of Th1 cytokines [20]. Exposure to nitrogen dioxide (NO_2_) at domestic concentrations causes impairment of the skin barrier function in subjects with AD [21]. Airborne particulate matter (PM) has also been identified as a risk factor for deteriorating skin condition in patients with AD [22]. These findings suggest that indoor air pollutants play a key role in the development and aggravation of allergic diseases such as AD [4].

To understand how home remodeling and FA may be synergistically related to AD, it is important to understand their relationship. Patients with AD are known to have other atopic diseases, such as IgE mediated food allergy [11]. Böhme et al. reported that 27% of patients with AD patients were sensitized to food allergens at 2 years of age, including egg (21%), peanuts (15%), milk (8%), and cod positive (2%) [23]. In a study performed in the United Kingdom (*N* = 1,402 children), egg sensitization on SPT was significantly associated with AD (OR 9.53, 95% CI 2.40–37.82, *P* < 0.05) [24]. Researchers also described that the process of recognition of food allergens through antigen-presenting cells in the eczematous skin may serve as an important mediator of food sensitization and FA [25].

Researchers have demonstrated that the development of FA due to exposure to indoor pollutants may be related to home remodeling [26, 27]. Shiue found that urinary arsenic, heavy metal, and phthalate concentrations are associated with peanut, egg, milk, and shrimp sensitizations [26]. Stelmach et al. reported that maternal exposure to phthalate during pregnancy increased the risk for FA [27].

Several studies have provided helpful information related to the synergistic effect of FA and home remodeling on the prevalence of AD [28, 29]. In particular, filaggrin (FLG) is an important protein in the skin and plays an important role in maintaining the integrity of the skin barrier [28]. FLG is associated with increased FA and several diseases with barrier dysfunction, such as* ichthyosis vulgaris *and AD [28]. In one study of patients with AD and the FLG gene variant, the higher skin permeability allowed the absorption of phthalate through the skin, resulting in higher urine phthalate metabolite levels than the control group [29]. This weakened skin barrier may lead to an increased absorption of indoor pollutants, such as phthalate, through the skin, aggravating eczematous skin lesions [29].

Efforts are needed to decrease the prevalence of AD related to home remodeling or reduce symptomatic deterioration in patients with AD. The common and basic way to improve indoor air quality is frequent cleaning, vacuuming, and ventilation [30]. The use of environmentally friendly materials in wallpaper and flooring should also be considered [6]. Lee et al. reported that there is a close relationship between the construction year of the house or moving to a newly constructed building within 1 year and formaldehyde level [31].

We acknowledge that this study has several limitations. We used a cross-sectional study design and gathered data via a questionnaire; therefore, we cannot infer a causal relationship. There is also the possibility of biases due to the methodology used. However, we conducted this study in a regional area intentionally and analyzed questionnaires and laboratory findings to reveal, for the first time in a Korean population, the relationship between allergic diseases and indoor pollution. Therefore, our study may be used as a representative study to increase awareness of the seriousness of indoor pollution among Korean children. Cohort studies are needed to confirm our findings.

Altogether, we found that home remodeling and FA history are independent risk factors for AD in children. We also observed a synergistic effect between home remodeling and FA in increasing the risk for moderate-to-severe AD. To prevent and manage the development of AD, further studies are needed to clarify how indoor pollutants interact with food allergens in the development of AD with more objective measures, including urine and blood samples, as well as measurements of the concentration of indoor air pollutants.

## Figures and Tables

**Table 1:** Demographic and clinical characteristics of subjects.

Characteristics	
*Number*	4,111
*Age (years), mean ± SD *	8.00 ± 1.85
*Sex (Boy : Girl)*	2,121:1,980 (51.7%:48.3%)
*BMI*	17.44 ± 2.83
*Parental history of allergic diseases*	2,312/4,111 (56.2%)
Parental history of asthma	203/4,111 (4.9%)
Parental history of allergic rhinitis	2,075/4,111 (50.5%)
Parental history of AD	400/4,111 (9.7%)
Parental history of FA	346/4,111 (8.4%)
*Environmental tobacco smoking*	1,475/4,041 (36.5%)
*Lifetime home remodeling*	1,146/4,014 (28.6%)
*Moving to new home in infancy*	1,054/3,299 (31.9%)
*Educational degree of mother*	
≤High school graduate	795/3,984 (20.0%)
≥University graduate	3,189/3,984 (80.0%)
*Parental economic status (monthly income)*	
Low (<2,697 USD)	439/3,860 (11.4%)
Middle (2,697–5,393 USD)	1,700/3,860 (44.0%)
High (≥5,393 USD)	1,721/3,860 (44.6%)
*Biomarkers*	
Eosinophil (%), mean ± SD	3.85 ± 2.97
*Atopy* ^*∗*^	297/575 (51.7%)

SD: standard deviation; BMI: body mass index; AD: atopic dermatitis; FA: food allergy; USD: United States of America dollar. ^*∗*^Defined as at least 1 positive skin prick test (allergen and histamine wheal diameter > 3 mm).

**Table 2:** Prevalence of AD.

	Number	Prevalence, %
Lifetime symptoms	775/4,111	18.9
Symptoms in the past 12 months	588/4,111	14.3
Lifetime diagnosis	1,240/4,111	30.2
Treatment in the past 12 months	403/4,111	9.8
Current AD^*∗*^	483/4,111	11.7
Moderate-to-severe AD^†^	38/578	6.6

AD: atopic dermatitis; SCORAD: scoring atopic dermatitis. ^*∗*^Defined as lifetime diagnosis together with symptoms in the past 12 months in the questionnaire; ^†^578 participated in SCORAD testing. Moderate-to-severe AD was defined as a SCORAD score > 25.

**Table 3:** Risk factors for lifetime diagnosis of AD.

Risk factors	*N* (%)	
	435/4,111 (10.6%)	
	aOR^*∗*^ (95% CI)	*P* value
*Demographic factors*		
Age (older)	0.96 (0.85–1.07)	0.431
Sex (girl)	**1.69 (1.10–2.58)**	**0.016**
BMI	1.03 (0.95–1.11)	0.514
Educational status of the mother (≥university graduate)	1.61 (0.99–2.62)	0.053
Economic status (higher monthly income)	1.12 (0.84–1.48)	0.436
*Personal factors*		
Lifetime diagnosis of FA	**3.95 (2.00–7.83)**	**<0.001**
Lifetime diagnosis of asthma	**3.38 (1.72–6.67)**	**<0.001**
Lifetime diagnosis of allergic rhinitis	**2.37 (1.57–3.60)**	**<0.001**
Breast milk feeding	1.55 (0.94–2.58)	0.089
Premature birth	1.86 (0.89–3.91)	0.101
Delivery (Cesarean section)	0.96 (0.63–1.46)	0.858
*Genetic factors*		
Parental history of allergic diseases	3.22^†^** (2.04–5.10)**	**<0.001**
Parental history of AD	2.67^†^** (1.47–4.83)**	**0.001**
Parental history of asthma	2.16 (0.90–5.21)	0.087
Parental history of allergic rhinitis	**2.46 (1.60–3.79)**	**<0.001**
Parental history of FA	**2.35 (1.31–4.24)**	**0.004**
*Environmental factors*		
Dog ownership	0.61 (0.18–2.10)	0.432
Cat ownership	1.48 (0.23–9.51)	0.681
Day care attendance before 1 year old	0.66 (0.18–2.39)	0.523
Older siblings	0.76 (0.51–1.13)	0.174
Home remodeling, ever	1.37 (0.89–2.12)	0.151
Home remodeling in infancy	0.86 (0.16–4.78)	0.864
Home remodeling in the past 12 months	**3.40 (1.43–8.09)**	**0.006**
Moving to new home in infancy	0.98 (0.62–1.56)	0.944
*Biomarkers*		
Eosinophil > 4%	2.37^**∗∗**^** (1.54–3.62)**	**<0.001**
Eosinophil 4th quartile (>5.0%)	2.49^**∗∗**^** (1.57–3.94)**	**<0.001**

The data were calculated by logistic regression multivariate analysis. aOR: adjusted odds ratio; CI: confidence interval; BMI: body mass index; FA: food allergy; AD: atopic dermatitis. ^*∗*^aOR was adjusted by age, sex, BMI, parental history of AD, familial income, and eosinophil. ^†^aOR was adjusted by age, sex, BMI, familial income, and eosinophil. ^*∗∗*^aOR was adjusted by age, sex, BMI, parental history of AD, and familial income. Significant aOR and *P* values are in bold.
